# Psychiatric Disorders among the Military in West Africa: A Systematic Narrative Review

**DOI:** 10.3390/bs11100138

**Published:** 2021-10-11

**Authors:** Winifred Asare-Doku, Gordon Maanianu Donnir, Kenneth Ayuurebobi Ae-Ngibise, Jennifer Peprah, Kofi Awuviry-Newton, Francis Acquah

**Affiliations:** 1 National Drug and Alcohol Research Centre, Faculty of Medicine, University of New South Wales, Sydney, NSW 2031, Australia; 2 School of Medicine and Public Health, College of Health, Medicine and Wellbeing, The University of Newcastle, Callaghan, NSW 2308, Australia; gordon.donnir@uon.edu.au (G.M.D.); kenneth.aengibise@uon.edu.au (K.A.-N.); jennifer.peprah@uon.edu.au (J.P.); 3 Kintampo Health Research Centre, Kintampo P.O Box 200, Bono East Region, Ghana; 4 African Health and Ageing Research Centre (AHARC), Winneba, Ghana; kofi.awuvirynewton@uon.edu.au; 5 Positive Wellness Recovery Centre, Melbourne, VIC 3076, Australia; francis@pmhp.com.au

**Keywords:** conflict, military, deployment, psychiatric disorders, mental health, Africa

## Abstract

(1) Background: Military combat impacts the mental health of veterans and active military personnel. Although various studies, the majority of which are from Westernized countries, have provided insight into how combat causes psychiatric disorders, such as post-traumatic stress disorder (PTSD), depression, and anxiety among veterans, there appears to be scant published literature on psychiatric disorders among military personnel in the West African region. It is important to contextually understand the psychiatric disorders among military personnel in this region who may be faced with similar vulnerabilities to their counterparts from Western cultures. (2) Methods: This study follows the Preferred Reporting Items for Systematic reviews and Meta-Analyses (PRISMA) guidelines. Studies were included if they were published in English between January 2010 and January 2021 and assessed mental health problems or psychiatric disorders among soldiers in West Africa. (3) Results: All three included studies were from Nigeria. High prevalence rates of substance and alcohol use were reported. (4) Conclusions: This review highlights the need for more research in this population as well as identifying the intervention needs of the soldiers and their implications.

## 1. Introduction

Studies have indicated that approximately 50% of people in military combat have developed mental health problems including post-traumatic stress disorder (PTSD), anxiety, and depression [1]. PTSD is noted to affect military and veteran populations more than other psychiatric disorders [2]. PTSD is a disorder that develops in response to a traumatic event and the primary mental health of concern among the military [3]. In some studies, PTSD rates are higher among the military than the general population [4].Chronicity occurs if the psychopathology that causes these difficulties is not addressed, leading to considerable social impairment, marital dysfunction, employment instability, suicide, drug misuse, and aggressive conduct [5]. Chronic musculoskeletal conditions, mood disorders, and PTSD have been established as primary areas of focus in prevention and control of disability in servicemen [6].

Deployment to military activities has a detrimental impact on deployed military personnel’s mental health [7]. It has consistently been documented that deployment has a negative impact on all mental health categories—PTSD, depression, drug abuse/dependence, suicide, and other mental illnesses—in evaluations done more than 24 months after returning from deployment [8]. One systematic review found that military personnel reported increased levels of anxiety, psychological distress, PTSD, and depression but less alcohol misuse [9]. Other previous reviews examined the psychiatric disorders in veterans of the Persian Gulf War of 1991 between 1990 and 2001 and findings showed increased prevalence of PTSD and common mental disorders but little evidence concerning alcohol misuse or dependence in all the included studies [10]. Another review examined the relationship between mental health and deployment length and found that an increase in deployment length led to increased adverse health effects [11]. No studies on Africa were included in these reviews. Previous studies have reported a significant risk of mental problems among military personnel on combat duty as well as difficulties in receiving mental health services because of the stigma associated with psychiatric care [12,13,14]. The deployment duration, the number of previous deployments, and exposure to combat-related trauma all impact the mental health of the military [15]. Marital distress has been reported as one of the consequences among active-duty military personnel as well as the need for couple-based intervention to prevent or address psychopathology in military personnel [16]. Psychiatric problems have also been reported to moderate the relationship between insomnia and cognitive disorders in military soldiers [17]. Although the majority of these studies are from Western countries, they provide insight into the prevalence of psychiatric disorders in this population.

West Africa is host to several national threats, including civil wars, political unrest, Islamic extremism, terrorist groups, armed criminal activities, illicit drugs trade, and pandemics [18,19]. Some countries in West Africa were classified by the World Bank as hosting fragile and conflict-affected situations including Burkina Faso, Guinea-Bissau, Mali, Niger, and Nigeria [20]. Evidence shows that the United States military, British, and French troops are usually deployed to the West African region to provide military support and peacekeeping [21,22]. Combat-related trauma during deployment is associated with PTSD, suicide, anxiety, substance misuse, and depression which directly impact physical health, homelessness, aggression, violence, and criminality [2]. While there are quite extensive reviews on the impact of deployment on military personnel, only a few have been done in West Africa. The few that have been done have mostly focused on deployments following a natural disaster such as a disease outbreak and were done on US military personnel who were deployed to West Africa during the Ebola outbreak [23,24]. Therefore, it is important to explore and investigate within the context of the West African region the prevalence of psychiatric disorders among West African military personnel and the psychiatric implications of military combat deployment to inform policy and intervention programs for this population given the history of wars and armed criminal activities in the sub-region.

This review seeks to highlight the dearth of research in this population in West Africa by systematically identifying relevant original research and analyzing their report of psychiatric morbidity among military personnel who have been deployed to combat zones, as well as illuminating intervention needs to inform policy. Additionally, such a review seeks to advocate for more research in this population to further understand the types of mental disorders to inform specific intervention programs. The paucity of literature about the psychiatric outcomes of military combat in the African region, particularly in West Africa, justifies a need to employ a narrative review to establish a theoretical framework for future focused studies [25,26].

## 2. Materials and Methods

### 2.1. Search Strategy

This review followed the Preferred Reporting Items for Systematic reviews and Meta-Analyses (PRISMA) guidelines [27]. A narrative review was systematically conducted to understand the psychiatric disorders among active military personnel according to the PRISMA guidelines. The following electronic databases were searched: PubMed, MEDLINE, PsycINFO, EMBASE, and PsycExtra. A comprehensive search was conducted by searching keywords including “mental health”, “psychiatric disorders”, “mood disorders”, “PTSD”, “soldier”, “military”, “military personnel”, and “West Africa” in the five electronic databases. In addition, a manual review of the reference list of included papers were searched. Two authors independently screened the titles and abstracts of identified studies and duplicates were removed. Studies considered eligible for full text screening were retrieved for full review.

### 2.2. Inclusion and Exclusion Criteria

Studies were included if they were published in English between January 2010 and January 2021. This timeframe was selected because authors sought to examine current studies conducted in this field. Original studies that measured mental health problems or psychiatric disorders among military personnel in West Africa were included. All types of military personnel were eligible including the air force, armed forces, navy, special forces, etc. Studies that did not meet these criteria were excluded. The search was limited to peer-reviewed published articles.

### 2.3. Data Extraction

**Figure 1:**
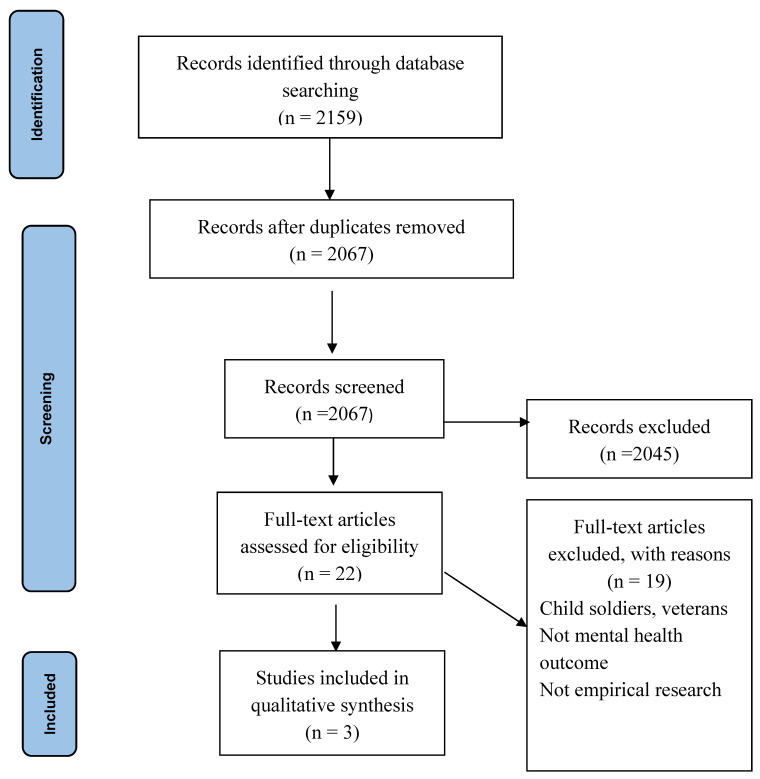
PRISMA flowchart- screening processes for narrative synthesis.

Titles of the abstracts were screened for appropriateness by two reviewers (W.A.D. and J.P.). Both reviewers reviewed the abstracts with close reference to the inclusion and exclusion criteria to eliminate studies not meeting the selection criteria. Papers were included if they reported on military personnel with a psychiatric or mental disorder, self-report of military personnel having a psychiatric or mental disorder either self-reported or measured with an instrument and were reported in English. Differences between reviewers’ results were resolved by discussion and in consultation with a third reviewer (G.D.). Full texts of articles which met criteria were retrieved. Authors manually went through the reference lists of the identified papers and titles of references were searched to retrieve abstracts and full texts to maximize the search. Those meeting eligibility criteria were included. A full summary of included studies is reported in Figure 1.

Data extracted from each paper satisfying the inclusion criteria was entered into a summary table in the following categories: authors, year of publication, country, study design, and key findings. No ethical approval was required as the review is based on published data. Data were extracted from the full-text references into a Microsoft Excel document. Narrative synthesis was used to identify the prevalence of psychiatric disorders among the military personnel.

### 2.4. Risk of Bias

The Joanna Briggs Institute for cross-sectional studies was used to assess the methodological quality of a study and to determine the extent to which a study has addressed the possibility of bias in its design, conduct, and analysis. The appraisal tool consists of eight questions answered as “yes”, “no”, “unclear”, or “not applicable” [28]. All items answered “yes” were considered low risk of bias and items answered “no” or “unclear” were considered a high risk of bias.

## 3. Results

The PRISMA flowchart screening processes for narrative synthesis is shown in Figure 1.

In total, 2159 articles were identified in the initial search. Duplicate studies were removed, and further screening was completed. Only three papers satisfied the inclusion criteria, which were all from Nigeria (See Table 1). Males were overrepresented, most were less than 30 years old, married, and had secondary level education. All three included studies using a descriptive cross-sectional study.

### 3.1. Description of the Included Studies

The median age was 32 years with majority of the participants being men (90.1%) and married (70.4%) [29,30]. However, in the Lasebikan and Ijomanta [31] study, the median age of the respondents was 38 years. The military personnel included primary job description was peacekeeping and internal security operations, both within and outside Nigeria. All three included studies were classified as low risk of bias.

Narrative synthesis is presented in relation to the aim of the study: determining the prevalence of psychiatric disorders among military personnel in Africa. The synthesis of the evidence resulted in two main themes: prevalence of psychiatric disorders among deployed military personnel and predictors of psychiatric disorders. The findings, all together, echo the understanding of the extent to which psychiatric dsiorders prevail and can affect the military personnel in the sub-Saharan Africa.

### 3.2. Prevalence of Psychiatric Disorders over 12-Month Period

The study reported whether the deployed military personnel had ever used, abused, and depended on cannabis in their operations. According to [31], the 12-month prevalence of non-medically prescribed opioid use was 6.7% and NMPOU is 3.6%. The prevalence was 53.6% with alcohol, a higher proportion among all other disorders [30]. The prevalence of 12-month cannabis use was 6.8% whereas the cannabis use disorder was 2.2% in [29]. Tobacco use and cannabis use were predictive of NMPOU, while 12-month alcohol use disorder, 12-month nicotine dependence, and 12-month cannabis use disorder were predictors of NMPOU disorder.

### 3.3. Prevalence of Lifetime Psychiatric Disorders

The included study revealed psychiatric lifetime implications on deployed military personnel; however, there are varying degrees of occurrence. In Lasebikan and Ijomanta [29], lifetime cannabis use was 13.5%, lifetime cannabis abuse was 4.9%, lifetime cannabis dependence was 0.9%, and lifetime cannabis use disorder was 5.8%. A similar psychiatric disorder prevalent among deployed military personal was non-medically prescribed opioid use and disorder [31]. Among the lifetime psychiatric disorders prevalent in deployed military personnel, the prevalence of alcohol use was high, representing 76.0% [30]. The prevalence of binge drinking among lifetime alcohol users was 6.7% [30]. Findings also showed that lifetime cannabis users were significantly associated with participants who had ever received disciplinary action, had ever been deployed to operational areas, or had ever been injured in combat, while 12-month cannabis use was significantly associated with ever having received disciplinary action or ever having been injured in combat.

## 4. Discussion

The aim of the review was to explore the prevalence of psychiatric disorders among military personnel in the West African region and the psychiatric implications of military combat. Findings showed that the deployed military personnel used, abused, and depended on cannabis in their operations. According to Lasebikan and Ijomanta [31], the 12-month prevalence of non-medically prescribed opioid use (NMPOU) was 6.7% and NMPOU disorder was 3.6%. The 12-month prevalence of alcohol was 53.6% [30]. The prevalence of 12-month cannabis use was 6.8% whereas the cannabis use disorder was 2.2% among the soldiers [29].

Findings by Lasebikan and Ijomanta [29] showed that lifetime cannabis use was 13.5%, lifetime cannabis abuse was 4.9%, lifetime cannabis dependence was 0.9%, and lifetime cannabis use disorder was 5.8%. A similar substance use disorder that was prevalent among deployed military personal was the non-medically prescribed opioid use and disorder [31]. These findings are supported by evidence from the United States military personnel deployed to Iraq and Afghanistan. Personnel were more likely to develop a drug use disorder and alcohol use disorder [32]. Among the lifetime psychiatric disorders prevalent in deployed military personnel, the prevalence of alcohol use was high, representing 76% [30]. The prevalence of binge drinking among lifetime alcohol users was 6.7% [30]. Substance and alcohol use may be used as a coping method to alleviate combat-related trauma, but it is maladaptive. The outcomes of risky drinking and substance use in this group is poorer mental and physical health outcomes, poor social functioning, and reduced capacity [33]

The predictors of cannabis use and alcohol use were having elementary education, ethnicity (Hausa/Fulani), minority tribes, history of disciplinary action, history of deployment to operational areas, and history of injury in combat [29,30]. Regarding non-medically prescribed opioid use and disorder, the associating factors were 12-months tobacco use and 12-months cannabis use disorder [31].

Drawing on the evidence from the narrative review, deployed military personnel used, abused, and depended on cannabis in their operations. Similar findings were reported in a systematic review study with US veterans; high prevalence rates of substance and alcohol use disorders were found [34]. The findings in the review, however, did not examine other psychiatric disorders including PTSD. In contrast, other reviews found less alcohol misuse among military personnel [9]. Other previous reviews examined the psychiatric disorders in veterans showing increased prevalence of PTSD and common mental disorders but little evidence concerning alcohol misuse or dependence in all the included studies [10]. Another review examined the relationship between mental health and deployment length and found that an increase in deployment length led to increased adverse health effects [11]. These studies found limited evidence for alcohol and substances and no studies on Africa were included in the review.

It is known that substance and alcohol use are significant problems in the military for reasons such as coping, boredom, loneliness, and lack of recreational activities, hence it is not surprising that high prevalence rates were found in the three studies included in the review [35]. Drinking appears to be an acceptable culture in military service, because from 1982, active duty military personnel were legally able to use alcohol on base, regardless of the legal drinking age off-base [36]. This drinking culture may have contributed to normalizing this among military personnel. The three studies focused on specific components of the Composite International Diagnostic Interview (CIDI) questionnaire. These components of the CIDI were drug and alcohol section. The CIDI is a comprehensive structured interview to assess mental disorders according to the definition of the ICD-10 and DSM-IV [37]. Because the other disorders were not measured in their study, it is difficult to ascertain whether there would have been reports of psychiatric disorders. Based on evidence, it is probable that psychiatric disorders might be present but were not assessed. Similar prevalence rates of substance use disorders have been reported in Germany [38]. Higher rates of alcohol misuse have been reported in the UK armed forces [39]. According to Lasebikan and Ijomanta [31], the 12-month prevalence of non-medically prescribed opioid use (NMPOU) was higher than that of NMPOU disorder. The prevalence was also greater for alcohol dependence as a coping mechanism. Lasebikan and Ijomanta [29] identified that lifetime cannabis use was higher compared to lifetime cannabis abuse. Additionally, lifetime cannabis dependence was lower than lifetime cannabis use disorder. These findings reflect those of Murdoch et al. [8], who stated that up to 24 months after service, veterans are impacted by vulnerabilities including drug and alcohol use, abuse, and disorder.

### 4.1. Limitations and Recommendations

The systematic review protocol was not registered in PROSPERO. This study was limited to three articles, all from one country, Nigeria—this was the first study among the military population. Only English language papers were included in the review. Meta-analysis was not conducted because the studies were with the same sample. This review shows a huge gap; further research is needed to ascertain the prevalence of psychiatric disorders among the military population. To inform policy interventions for treatment and rehabilitation and prevention for the military, it is important to understand the extent of psychiatric disorders prevalent in this population. In addition, all three studies reported substance and alcohol use among the soldiers with no data on the extent of psychiatric disorders in this population.

### 4.2. Conclusions

This review has shown limited original research in investigating psychiatric disorders among military personnel in the West African. The review, therefore, has highlighted the severe dearth of evidence of psychiatric disorders in this population and therefore a call for West African governments and research funding organizations to invest in original research in the region to inform policy and intervention strategies. Again, the included studies all came from Nigeria and only reported on substance use among military personnel without any report on intervention programs post-deployment. This reveals gaps to prioritize future research in this population.

## Figures and Tables

**Table 1:** Summary of included studies.

Author	Study Aim	Country	Method	Study Design	Sample Size	Risk of Bias	Key Finding
Lasebikan, V. O. and Ijomanta, I. N. (2018)	To determine the prevalence of both lifetime and 12 months and cannabis use disorder and their correlates among a military population.	Nigeria	A multi-stage stratified systematic sampling method was used to sample participants.	Descriptive cross-sectional study	223	Low	Higher lifetime prevalence of cannabis use; 12-month cannabis use was entirely confined to men; cannabis use was associated with past disciplinary action in the workplace.
Lasebikan, V. O. and Ijomanta, I. N. (2019)	To ascertain 12-month prevalence of non-medically prescribed opioid use and non-medically prescribed opioid use disorder among military population.	Nigeria	A multi-stage stratified systematic sampling method was used to sample participants.	Descriptive cross-sectional study	223	Low	The 12-month prevalence of non-medical prescription opioid use was 6.7% and non-medical prescription opioid use disorder, 3.6%. Non-medical prescription opioid use was more common among those who ever got injured during combat. Of the participants who had a combat injury, 8% had lifetime use of prescription opioids and developed either abuse or dependence.
Ijomanta, I. N. and Lasebikan, V. O. (2016)	To establish the lifetime and 12 months prevalence of alcohol use and alcohol use disorders as well as the profile of problems associated with the diagnoses of alcohol use disorders among a military population.	Nigeria	A multi-stage stratified systematic sampling method was used to sample participants.	Descriptive cross-sectional study	223	Low	Prevalence of lifetime alcohol use was 76%, 12 months prevalence was 54%, and frequent binge drinking was reported in 7% of respondents. Alcohol use is more highly prevalent among the military personnel than the general population.

## Data Availability

The review used existing research data.
